# Comparative evaluation of the efficiency of the BG-Sentinel trap, CDC light trap and Mosquito-oviposition trap for the surveillance of vector mosquitoes

**DOI:** 10.1186/s13071-016-1724-x

**Published:** 2016-08-12

**Authors:** Yiji Li, Xinghua Su, Guofa Zhou, Hong Zhang, Santhosh Puthiyakunnon, Shufen Shuai, Songwu Cai, Jinbao Gu, Xiaohong Zhou, Guiyun Yan, Xiao-Guang Chen

**Affiliations:** 1 Key Laboratory of Prevention and Control for Emerging Infectious Diseases of Guangdong Higher Institutes, Department of Pathogen Biology, School of Public Health and Tropical Medicine, Southern Medical University, Guangzhou North Avenue No.1838, Guangzhou, 510515 China; 2 Program in Public Health, School of Medicine, University of California, Irvine, USA; 3 Department of Vector Control, Centers for Disease Control and Prevention of Guangdong Province, Guangzhou, China

**Keywords:** BG-Sentinel trap, CDC light trap, Mosq-ovitrap, *Aedes albopictus*, *Culex quinquefasciatus*, *Anopheles sinensis*, Surveillance

## Abstract

**Background:**

The surveillance of vector mosquitoes is important for the control of mosquito-borne diseases. To identify a suitable surveillance tool for the adult dengue vector *Aedes albopictus*, the efficacy of the BG-Sentinel trap, CDC light trap and Mosquito-oviposition trap (MOT) on the capture of vector mosquitoes were comparatively evaluated in this study.

**Methods:**

The capture efficiencies of the BG-Sentinel trap, CDC light trap and Mosquito-oviposition trap for common vector mosquitoes were tested in a laboratory setting, through the release-recapture method, and at two field sites of Guangzhou, China from June 2013 to May 2014. The captured mosquitoes were counted, species identified and compared among the three traps on the basis of species.

**Results:**

In the release-recapture experiments in a laboratory setting, the BG-Sentinel trap caught significantly more *Aedes albopictus* and *Culex quinquefasciatus* than the CDC light trap and Mosquito-ovitrap, except for *Anopheles sinensis*. The BG-Sentinel trap had a higher efficacy in capturing female rather than male *Ae. albopictus* and *Cx. quinquefasciatus*, but the capture in CDC light traps displayed no significant differences. In the field trial, BG-Sentinel traps collected more *Aedes albopictus* than CDC light traps and MOTs collected in both urban and suburban areas. The BG-Sentinel trap was more sensitive for monitoring the population density of *Aedes albopictus* than the CDC light trap and MOT during the peak months of the year 2013. However, on an average, CDC light traps captured significantly more *Cx. quinquefasciatus* than BG-Sentinel traps. The population dynamics of *Cx. quinquefasciatus* displayed a significant seasonal variation, with the lowest numbers in the middle of the year.

**Conclusions:**

This study indicates that the BG-Sentinel trap is more effective than the commonly used CDC light trap and MOT in sampling adult *Aedes albopictus* and *Culex quinquefasciatus*. We recommend its use in the surveillance of dengue vector mosquitoes in China.

**Electronic supplementary material:**

The online version of this article (doi:10.1186/s13071-016-1724-x) contains supplementary material, which is available to authorized users.

## Background

Mosquitoes are vectors of various human and animal diseases, such as malaria, filariasis, dengue fever, Chikungunya fever, Japanese encephalitis, and yellow fever [1]. Vector control is an essential component and sometimes is the only effective way (e.g. for dengue fever) to block or reduce the transmission of these diseases [2–5]. Using a sensitive and efficient surveillance tool to monitor the species composition and population dynamics of local mosquitoes is the most important step in developing and implementing appropriate strategies to control vector populations.

At present, there are already several methods or techniques to survey the population and density of vector mosquitoes [6–13]. However, varied efficacies have been reported for different types of traps [14, 15]. Currently, the commonly used methods in surveillance programmes to collect adult mosquitoes in China include the Centre for Disease Control and Prevention (CDC) light trap, Mosquito-oviposition trap (MOT) and human landing catches. CDC light traps are the most commonly used method for the surveillance of mosquito populations, and many previous reports have shown that it is effective in capturing *Culex* and *Anopheles* but not *Ae. albopictus* [16, 17]. *Aedes albopictus* is a daytime-biting (from dawn to dusk) mosquito species and is the primary vector of dengue fever in China [18]. A previous report has indicated that the CDC light trap is not efficient for the surveillance of *Ae. albopictus* [19]. MOTs are primarily used to collect eggs and female adults of *Ae. albopictus*, but the efficacy in collecting adult mosquitoes is low [20]. Although the human landing catch is a very effective way to catch adult mosquitoes, especially *Aedes* mosquitoes [21, 22], it leads to serious ethical concerns associated with the use of humans as bait because of the potential risk of infection with dengue viruses. All these facts indicate the lack of a safe, standard and sensitive method for vector mosquito surveillance in China, especially for the dengue vector mosquito, *Ae. albopictus*.

In recent years, BG-Sentinel traps (BioGents Corporation, Regensbourg, Germany) have been used to collect *Aedes* (*Stegomyia*) mosquitoes such as *Ae. aegypti*, *Ae. albopictus*, and *Ae. polinesiensis* [23–26]. BGS traps can be used with a variety of mosquito attractants, e.g. CO_2_, BG-lure, and octanol, thereby making it a versatile tool for mosquito research and surveillance. Nevertheless, there is no report on evaluating the efficiency of the BG-Sentinel trap in comparison with other traps for the surveillance of dengue vector mosquitoes in China.

In this study, we evaluated the efficacy of the BG-Sentinel trap, CDC light trap and MOT traps in terms of mosquito captures by species in a laboratory setting as well as in the fields of Guangzhou, Guangdong province, China.

## Methods

### Description of study sites

Laboratory-based experiments were carried out at the Centre for Disease Control and Prevention of Guangdong Province, Guangzhou, China, and field trials were conducted from June 2013 to May 2014 in Tonghe and Liangtian of Guangzhou. Guangzhou is the largest city in Southern China, with a population of 12 million according to the 2012 census. Guangzhou has been the major region of dengue epidemic in China in recent years [27]. The annual average temperature is 21.6 °C, and rainfall is 1,983 mm. This climate is ideal for the development and reproduction of vector mosquitoes.

**Fig. 1:**
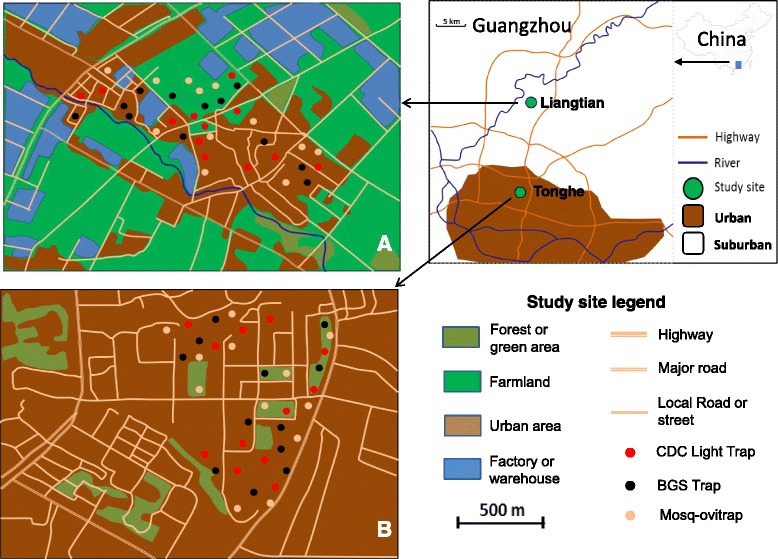
Map of the study areas and distribution of the traps in the first week of each month. **a** Liangtian (suburban area), **b** Tonghe (urban area). Twelve each of BGS Traps, CDC Light Traps and MOTs were used to survey the mosquito density in Tonghe and Liangtian

Tonghe (113°19′E, 23°11′N, 31 m above sea level, m.a.s.l.) is an urban area with a population density of > 3,000 people/km^2^. The land use includes primarily residential and commercial buildings, as well as public services facilities, such as schools and hospitals, filled with trees and grasses. Liangtian (113°23′E, 23°21′N, 25 m a.s.l.) is a suburban area with a population density of approximately 1,000 people/km^2^, and land use includes a mixture of residential, manufacturing, and farmland (Fig. 1).

### The traps

Three types of traps were tested: BGS traps (BioGents, Regensbourg, Germany) with BG-Lure (BioGents, GmbH, Regensbourg, Germany), CDC light traps (Lucky Star Environmental Protection Technology Co., Ltd., Wuhan, China), and MOTs (Southeast Industrial Co. Ltd, Guangdong, China).

### Experimental design

In the laboratory, a release-recapture experiment [28] was conducted in a standard room (3.2 × 2.9 × 2.75 m) to determine the collection efficiency of the three types of traps. Three species of mosquitoes, *Ae. albopictus*, *Cx. quinquefasciatus* and *An. sinensis,* were tested and all experimental mosquitoes were 3–5 days post-emergence at release. We conducted the study with groups of 200, 400, and 800 *Aedes albopictus* to evaluate if mosquito abundances affect the efficiency of the traps. Then we conducted the study with groups of 200 each for *Aedes albopictus*, *Culex quinquefasciatus* and *Anopheles sinensis* to evaluate the efficacies of different traps on different mosquitoes. It was reported that the mark-release-recapture rate is low in the field [29]. In all groups, we used a female to male ratio of 1:1. The mosquitoes were released in the room and three traps with one of each type were kept in the room for 24 h. After 24 h, mosquitoes collected in each trap were counted. The experiments were repeated eight times. The three traps were put diagonally in the room, to reduce design bias, the positions of traps were rotated in the room following a Latin square design after each replication. The laboratory environmental conditions were set at a temperature of 26 ± 1 °C, with a relative humidity of 60–80 % and a light and dark period of 12 h.

In the field trial, 12 of each of three types of traps were placed in the two study areas. In the urban area, we chose three locations for setting traps: a residential area, a public park, and a commercial district; in the suburban area, the locations were a residential area, a factory, and a garden. The distance between two traps was at least 30 m. Traps were placed in the same location for three consecutive days during the first week of each month; and they were shifted to different locations for another three days during the third week of each month. The adult mosquito populations were monitored continuously from June 2013 to May 2014. The CDC light traps were hung in trees 0.8 m above the ground, whereas the BGS traps and MOTs were placed on the ground. Every 24 h, as one trapping period, mosquitoes were collected and transported to the laboratory for species identification. The geographical coordinates of each sampling point were recorded using portable global positioning system (GPS) devices (Garmin eTrex H) (Fig. 1 and Additional file 1: Figure S1).

### Mosquito identification

Frozen mosquitoes were placed on a piece of white filter paper in a Petri plate on a chill table, and the species were identified morphologically under a stereo microscope using taxonomic keys [30].

### Statistical analysis

Differences in sex-specific captures among different traps and mosquito species under laboratory conditions were tested using generalized estimating equation (GEE) based Negative Binomial regression and Tukey’s *post-hoc* honestly significant difference (HSD) tests. Differences in population dynamics between the BGS trap and CDC light trap were compared using the GEE Negative Binomial regression. Data were square-root transformed before Tukey’s HSD test. Statistical analysis was performed using the JMP statistical software (JMP 9.0, SAS Institute Inc., USA) and R 3.0.1. Differences in sex ratio in field-captured mosquitoes between the BGS traps and CDC light traps were compared using the *χ*^2^-test or the Fisher exact test if any number was < 5.

## Results

### Laboratory study

**Fig. 2:**
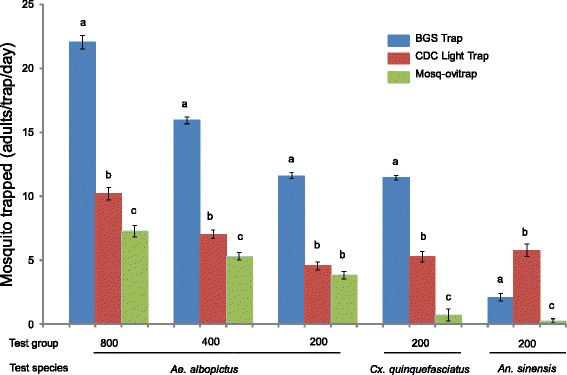
The effectiveness of the three kinds of traps in catching different species of mosquitoes in laboratory conditions. Comparative analysis of mean and standard error of *Aedes albopictus*, *Cx. quinquefasciatu*s and *An. sinensis* caught in different traps under laboratory conditions. Columns with different letters are significantly different from each other (*P* < 0.05). Mosquito abundance is square-root transformed, and values are the mean ± standard error

For *Ae. albopictus*, regardless of the number of individuals released, BGS traps increased capture rates almost 2-fold compared to CDC light traps. CDC light traps captured approximately 20 % more individuals than the MOTs (*P* < 0.001) (Fig. 2, Additional file 2: Table S1). Overall, regardless of population densities, the most efficient method of *Aedes albopictus* collection was the BGS trap, followed by CDC light traps and MOT. For *Cx. quinquefasciatus*, BGS traps caught twice the number of mosquitoes than CDC light traps, CDC light traps caught five times more mosquitoes than MOTs (*P* < 0.001) (Fig. 2, Additional file 2: Table S1). There was an exception for *Anopheles sinensis*. The CDC light traps captured almost three times more *An. sinensis* than the BGS traps and five times more than the MOTs (*P* < 0.001) (Fig. 2, Additional file 2: Table S1).

Table 1 illustrates the sex-specific capture by trap type and species. The BGS trap had a higher efficacy in capturing female rather than male *Ae. albopictus* and *Cx. quinquefasciatus* (Tukey’s HSD test, both *P* < 0.001), but there was no difference in the captures of male and female *An. sinensis* (Table 1). The CDC light trap showed no difference in the captures of male and female *Ae. albopictus* and *Cx. quinquefasciatus*, but for *An. sinensis* it captured more males than females (Tukey’s HSD test, *P* < 0.001). The MOT showed a higher efficiency in capturing male rather than female *Ae. albopictus*, with 5-fold more males captured than females (Tukey’s HSD test, *P* < 0.001), and there was no difference in the captures of male and female of both *Cx. quinquefasciatus* and *An. sinensis* (Tukey’s HSD test, *P* > 0.05) (Table 1).

**Table 1:** Sex differences among mosquitoes collected in the three mosquito traps in the laboratory

Traps	No. of release times	*Ae. albopictus*		*Cx. quinquefasciatus*		*An. sinensis*	
		Male	Female	Male	Female	Male	Female
BGS trap	8	7.59 ± 0.20^a^	8.78 ± 0.17^b^	7.50 ± 0.19^a^	8.65 ± 0.22^b^	1.26 ± 0.31^a^	1.53 ± 0.24^a^
CDC Light trap	8	3.30 ± 0.27^a^	3.15 ± 0.16^a^	4.09 ± 0.24^a^	3.29 ± 0.41^a^	4.77 ± 0.44^a^	3.23 ± 0.25^b^
Mosq-ovitrap	8	3.70 ± 0.31^a^	0.78 ± 0.24^b^	0.48 ± 0.36^a^	0.43 ± 0.31^a^	0.11 ± 0.13^a^	0.13 ± 0.13^a^

Rows with different letters are significantly different from each other (*P* < 0.05). Mosquito abundance is square-root transformed and values are the mean ± standard error

*Abbreviations*: *M* male, *F* female

### Field study

In our field study, a total of 864, 864 and 288 trap-periods with the BGS traps, CDC light traps and MOTs, respectively, were conducted from June 2013 to May 2014 in Tonghe. A total of 876, 864 and 288 trap-periods with the BGS traps, CDC light traps and MOTs, respectively, were performed from June 2013 to May 2014 in Liangtian. In Tonghe, a total of 27,174 mosquitoes were collected and identified by species. Among them, 7,600 were *Ae. albopictus*, 19,436 *Cx. quinquefasciatus*, 129 *Armigeres subalbatus*, and 9 *Toxorhynchites splendens* (Table 2). In Liangtian, a total of 52,649 mosquitoes were collected and identified by species: *Ae. albopictus* (2,636), *Cx. quinquefasciatus* (49,730), *Armigeres subalbatus* (336) and *Toxorhynchites splendens* (17) (Table 2). During the survey period, the MOT collected only 49 adults and 573 eggs of *Ae. albopictus* and 6 adults and 88 eggs of *Cx. quinquefasciatus* in Tonghe, with only 16 adults and 204 eggs of *Ae. albopictus* and 15 adults and 114 eggs of *Cx. quinquefasciatus* in Liangtian. Due to the small number of captures, the MOT data were not subjected to further analysis.

**Table 2:** Species and sex composition of mosquitoes collected in BG-Sentinel and CDC light traps in Guangzhou, China

Study area	Species	Trap method								Sex ratio difference (*P*-value)^b^
		BG-sentinel trap				CDC light trap				
		Total	Male	Female	F rate (95 % CI)^a^	Total	Male	Female	F rate (95 % CI)^a^	
Tonghe (urban)	Number of trap days	864				864				
	*Aedes albopictus*	6211	3641	2570	0.41 [0.40, 0.43]	1389	790	599	0.43 [0.41, 0.46]	0.23
	*Culex quinquefasciatus*	11,365	7588	3777	0.33 [0.32, 0.34]	8071	4758	3313	0.41 [0.40, 0.42]	< 0.0001
	*Armigeres subalbatus*	46	25	21	0.46 [0.32, 0.60]	83	49	34	0.41 [0.31, 0.52]	0.60
	*Toxorhynchites splendens*	1	1	0	n.a.	8	5	3	0.38 [0.14, 0.69]	n.a.
	Total No.	17,623	11,255	6368		9551	5602	3951		
Liangtian (suburban)	Number of trap days	876				864				
	*Aedes albopictus*	2181	1200	981	0.45 [0.43, 0.47]	385	195	190	0.49 [0.44, 0.54]	0.11
	*Culex quinquefasciatus*	18,442	12,517	5925	0.32 [0.31, 0.33]	31,288	16,233	15,055	0.48 [0.48, 0.49]	< 0.0001
	*Armigeres subalbatus*	152	91	61	0.40 [0.33, 0.48]	184	114	70	0.38 [0.31, 0.45]	0.70
	*Toxorhynchites splendens*	3	1	2	0.67 [0.21, 0.94]	14	10	4	0.29 [0.12, 0.55]	0.51
	Total No.	20,778	13,809	6969		31,871	16,552	15,319		

^a^F rate is defines as number of females over total

^b^Difference between the two trap-types. *χ* ^2^-test or Fisher exact test if any number is < 5

Over 99 % of the BGS trap and CDC light trap catches were either *Ae. albopictus* or *Cx. quinquefasciatus*. BGS traps captured 5-fold more *Ae. albopictus* than CDC light traps in both urban and suburban areas, whereas CDC light traps captured 25 % less *Cx. quinquefasciatus* than BGS traps in urban areas but 2-fold more in suburban areas (Table 2). In general, both BGS traps and CDC light traps captured significantly more males than females, regardless of species (Table 2). BGS traps captured significantly fewer female *Cx. quinquefasciatus* than CDC light traps (33 *vs* 41 %, *χ*^2^ = 124.4, *df* = 1, *P* < 0.0001) (Table 2).

### Population dynamics of the mosquitoes collected by the traps

**Fig. 3:**
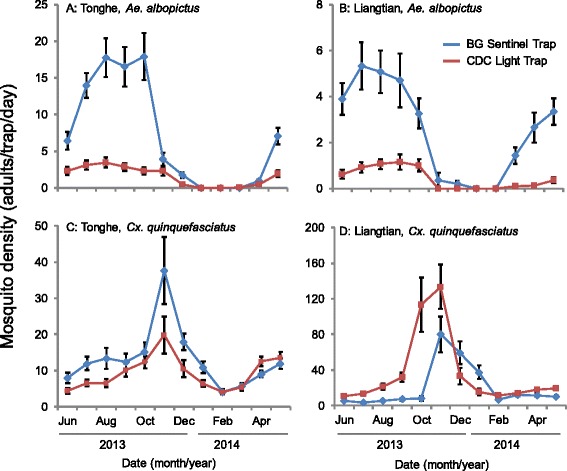
Population dynamics of *Aedes albopictus* and *Cx. quinquefasciatu*s collected by two traps in urban and suburban areas. **a **and **c** Tonghe (urban). **b** and **d** Liangtian (suburban). **a **and **b**: *Ae. albopictus*; **c** and **d**: *Cx. quinquefasciatus*. Values are the mean ± 95 % CI

The population dynamics of both *Ae. albopictus* and *Cx. quinquefasciatus* showed clear seasonal variation regardless of study site, mosquito species, and trap types (Fig. 3). The peak months varied depending on study area and species (Fig. 3). For *Ae. albopictus*, its captures were significantly more in urban than that in suburban area (*Z* = 15.91, *P* < 0.001), CDC light traps captured significantly less than BGS traps (*Z* = -25.13, *P* < 0.001) (Fig. 3, Table 3). The population dynamics of *Ae. albopictus* showed significant seasonal changes, with a peak collection likely in the middle of the year (Fig. 3, Table 3). In addition, in different areas, different trap types performed differently, likely due to the strong inter-site difference in population density (Table 3). In contrast, *Cx. quinquefasciatus* were captured year round with the highest densities in both BGS and CDC light traps observed from October to December (Fig. 3). The negative binomial regression analysis revealed that, *Cx. quinquefasciatus* density was significantly lower in urban than in suburban areas (*Z* = -5.24, *P* < 0.001), CDC light traps captured significantly more *Cx. quinquefasciatus* than BGS traps (*Z* = 13.85, *P* < 0.001) (Fig. 3, Table 3). The population dynamics of *Cx. quinquefasciatus* also showed significant seasonal variations, with the lowest catch likely in the middle of the year (Fig. 3, Table 3).

**Table 3:** Results of negative binomial regression analysis in the field study

Species	Coefficients	Estimate	Std. Error	*Z*-value	Pr(>|z|)
*Aedes albopictus*					
	(Intercept)	-6.11	0.18	-34.14	< 0.0001
	Site (urban)	0.85	0.05	15.91	< 0.0001
	TrapType (CDC)	-1.88	0.07	-25.13	< 0.0001
	Site (urban) * TrapType (CDC)	0.51	0.09	5.41	< 0.0001
	Month	2.07	0.05	42.20	< 0.0001
	Month*Month	-0.13	0.003	-41.18	< 0.0001
*Culex quinquefasciatu*s					
	(Intercept)	2.88	0.06	44.85	< 0.0001
	Site (urban)	-0.24	0.05	-5.24	< 0.0001
	TrapType (CDC)	0.62	0.05	13.85	< 0.0001
	Site(urban) * TrapType(CDC)	-0.88	0.06	-13.62	< 0.0001
	Month	-0.28	0.02	-13.48	< 0.0001
	Month*Month	0.03	0.002	19.86	< 0.0001

## Discussion

In both laboratory experiments and field surveillance tests, this study showed that BGS traps, compared to CDC light traps and MOTs, are highly efficient in capturing *Ae. albopictus* and *Cx. quinquefasciatus*. This work highlighted the efficiency of BGS traps and their further potential for implementation in the surveillance of vector mosquitoes in China.

The laboratory experiments indicated that BGS traps are more effective in capturing female adult mosquitoes of *Ae. albopictus* and *Cx. quinquefasciatus*. However, the field study showed that BGS traps have a higher efficacy in capturing males rather than females of *Ae. albopictus* and *Cx. quinquefasciatus*, which contradicts our original prediction, because BG lures consist of lactic acid, caproic acid and ammonium bicarbonate simulated human odorants that are thought to be more attractive to female mosquitoes. Similar results have also been reported from studies conducted in Brazil and Kenya [31, 32]. However, research from America showed that the ratio of male-to-female *Ae. albopictus* can vary seasonally, geographically, and in response to adulticidal treatments by BGS trap with lure [33, 34]. This phenomenon may be explained by mosquito behaviour. First, to mate with females, one shortcut for male mosquitoes is to locate potential hosts and remain nearby to increase their chances of encountering female mosquitoes [35–39]. It may be possible for male mosquitoes to recognize human odorants and use BGS traps as swarm markers. Secondly, the eclosion time for male and female mosquitoes is different. Usually, the male mosquitoes emerge first, and the females emerge afterwards. Thus, it is possible that for a period of time, the male is dominant in the environment, causing the biased attraction of BGS traps. Thirdly, male mosquitoes tend to stay in the wild, looking for nectar for food [36–39]. In this study, the traps were set in residential areas, public parks, commercial districts, factories, and gardens with open environmental spaces, where male mosquitoes tend to aggregate because of the abundant vegetation, whereas the females do not because of the lack of sufficient haematophagous hosts. This preference in distribution may also have caused the bias in catching.

The field surveys in this study showed that the species of mosquitoes in Guangzhou are *Ae. albopictus* and *Cx. quinquefasciatus*, a result consistent with those from previous reports [40, 41]. Analysis of the population dynamics of the mosquitoes showed that the population density of *Ae. albopictus* was much higher in the urban (Tonghe) than in the suburban area (Liangtian), which is consistent with the epidemic status of dengue in Guangzhou. Our results also showed that the monitoring of *Ae. albopictus* by BGS trap is more sensitive than that by CDC light traps, especially during the peak season. All of these data suggested that the BGS trap is a suitable surveillance tool for the dengue vector in China.

The different traps attract mosquitoes based on various mechanisms. BG-Sentinel traps use BG-lure (human odours) to attract host-seeking mosquitoes, CDC light traps use the light to attract phototaxis mosquitoes and Mosquito-oviposition traps use the water to attract oviposition-seeking mosquitoes respectively, therefore, these traps measure very different sub-populations of the mosquitoes at different stages in their life cycle. To reduce the bias that might be caused by the different physiological states of mosquitoes, these traps were placed in the same location for three consecutive days during the first week of each month and then shifted to different locations for another three days during the third week of each month for a total of 12 months. Although in different areas, different traps performed differently, likely due to the strong inter-site difference in population density and physiological states, the final summarized results of this study displayed that BGS traps are most efficient in capturing adult *Ae. albopictus* and *Cx. quinquefasciatus*, two species of vector mosquitoes most commonly distributed in the cities of China. Considering the role of *Ae. albopictus* in disease transmission, we recommend using the host-seeking based BGS traps as the surveillance tool of vector mosquitoes.

## Conclusion

We conducted the first comparative evaluation of the efficacy of BGS traps, CDC light traps and Mosq-ovitraps in the capture of common vector mosquitoes in a laboratory setting and in the field in Guangzhou, China. The results indicated that the BGS trap is an effective tool for the monitoring of urban vector mosquitoes and could be used in the surveillance of dengue fever in China.

## Additional files

Additional file 1: Figure S1. Distribution of the traps in the third week of each month. A. Liangtian (suburban area), B. Tonghe (urban area). Twelve each of BGS Traps, CDC Light Traps and MOTs were used to survey the mosquito density in Tonghe and Liangtian. (PDF 639 kb) Additional file 2: Table S1. Results of negative binomial regression in the laboratory study. (DOCX 15 kb)
