# Supplementary figures and images for: Comparative evaluation of the efficiency of the BG-Sentinel trap, CDC light trap and Mosquito-oviposition trap for the surveillance of vector mosquitoes
Source: Parasit Vectors. 2016 Aug 12;9:446. doi: 10.1186/s13071-016-1724-x (PMC4983048; doi:10.1186/s13071-016-1724-x)

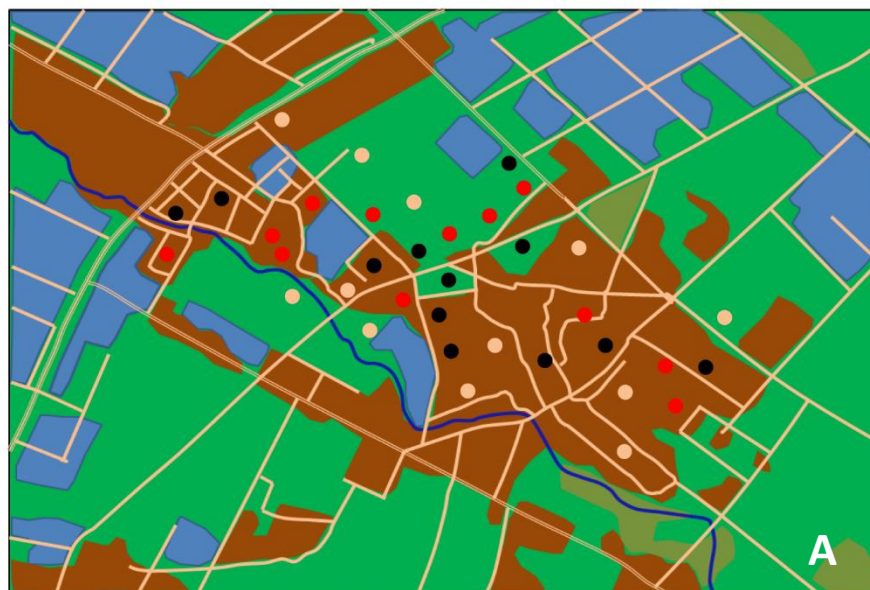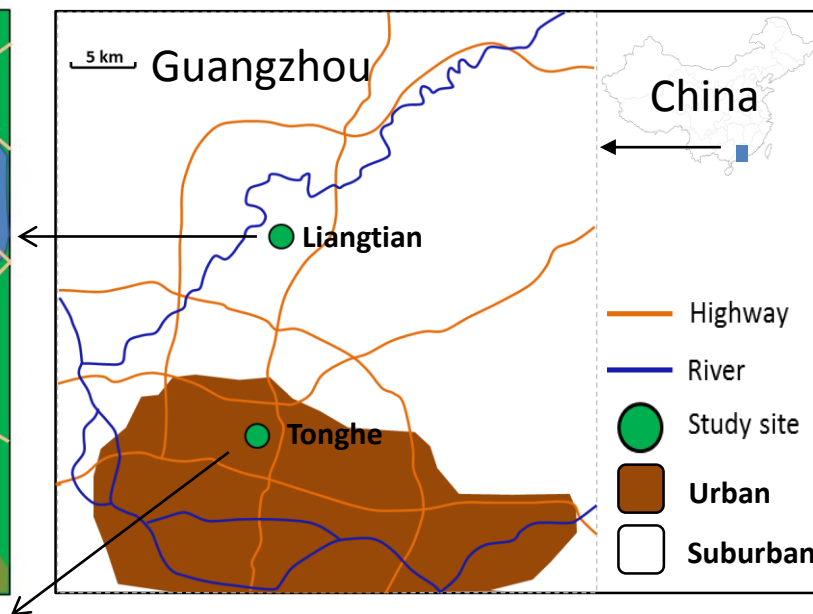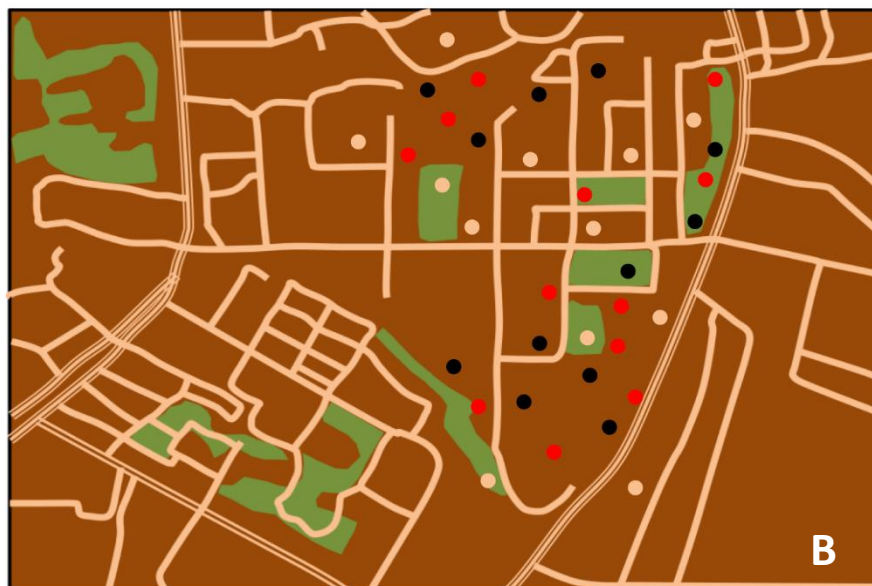

### Study site legend

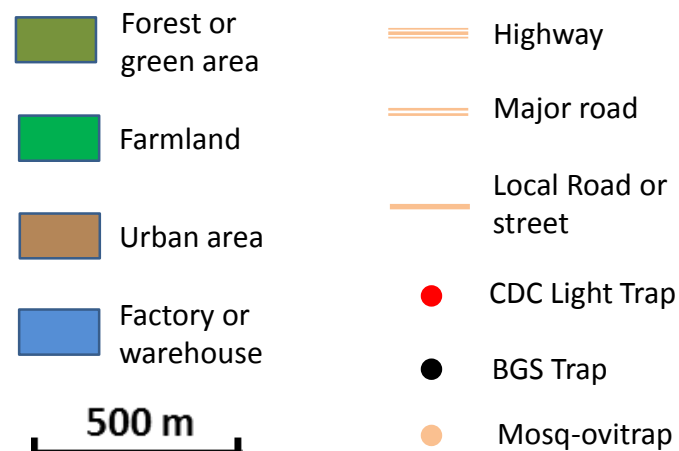

Supplement: Additional file 1: Figure S1. — Distribution of the traps in the third week of each month. A. Liangtian (suburban area), B. Tonghe (urban area). Twelve each of BGS Traps, CDC Light Traps and MOTs were used to survey the mosquito density in Tonghe and Liangtian. (PDF 639 kb) [file 13071_2016_1724_MOESM1_ESM.pdf]
