# Supplementary material for: Comparative evaluation of the efficiency of the BG-Sentinel trap, CDC light trap and Mosquito-oviposition trap for the surveillance of vector mosquitoes
Source: Parasit Vectors. 2016 Aug 12;9:446. doi: 10.1186/s13071-016-1724-x (PMC4983048; doi:10.1186/s13071-016-1724-x)
Supplement: Additional file 2: Table S1. — Results of negative binomial regression in the laboratory study. (DOCX 15 kb) [file 13071_2016_1724_MOESM2_ESM.docx]

**Table S1. Results of negative binomial regression in the laboratory study.**

| Species |  | Estimate | Std. Error | z value | Pr(>\|z\|) |
| --- | --- | --- | --- | --- | --- |
| Anopheles |  |  |  |  |  |
| N = 200 | Intercept | 1.61 | 0.22 | 7.35 | < 0.0001 |
|  | CDC Light Trap | 1.95 | 0.27 | 7.13 | < 0.0001 |
|  | MOT | -3.00 | 0.76 | -3.96 | < 0.0001 |
| Culex |  |  |  |  |  |
| N = 200 | (Intercept) | 4.88 | 0.13 | 36.25 | < 0.0001 |
|  | CDC Light Trap | -1.51 | 0.20 | -7.58 | < 0.0001 |
|  | MOT | -4.19 | 0.31 | -13.39 | < 0.0001 |
| Aedes |  |  |  |  |  |
| N = 200 | (Intercept) | 4.91 | 0.06 | 79.51 | < 0.0001 |
|  | CDC Light Trap | -1.84 | 0.11 | -16.44 | < 0.0001 |
|  | MOT | -2.17 | 0.12 | -17.85 | < 0.0001 |
|  |  |  |  |  |  |
| N = 400 | (Intercept) | 5.54 | 0.07 | 81.15 | < 0.0001 |
|  | CDC Light Trap | -1.62 | 0.10 | -15.91 | < 0.0001 |
|  | MOT | -2.17 | 0.11 | -20.46 | < 0.0001 |
|  |  |  |  |  |  |
| N = 800 | (Intercept) | 6.19 | 0.08 | 78.65 | < 0.0001 |
|  | CDC Light Trap | -1.53 | 0.11 | -13.29 | < 0.0001 |
|  | MOT | -2.19 | 0.12 | -18.38 | < 0.0001 |
